# Understanding the burden of ANCA-associated vasculitis

**DOI:** 10.1093/rheumatology/keaf520

**Published:** 2025-10-06

**Authors:** Bernhard Hellmich

**Affiliations:** Klinik für Innere Medizin, Rheumatologie, Pneumologie, Nephrologie und Diabetologie, Medius KLINIKEN Kirchheim-Teck & Nürtingen, Akademisches Lehrkrankenhaus der Universität Tübingen, Kirchheim unter Teck, Germany

**Keywords:** Anti-neutrophil cytoplasmic antibody, ANCA-associated vasculitis, AAV, disease burden, health-related quality of life, immunosuppression, glucocorticoids, avacopan

## Abstract

Anti-neutrophil cytoplasmic antibody (ANCA)-associated vasculitis (AAV) is a group of frequently relapsing systemic autoimmune disorders characterized by vasculitis-related organ damage and multiple comorbidities related to chronic inflammation. Advances in immunosuppression-based therapies for AAV have considerably improved remission rates, reduced the risk of relapse, and improved survival in patients with AAV. However, mortality remains high compared with the general population and the benefits of treatment are often offset by treatment-related comorbidities, organ damage and adverse effects, particularly infections. The aim of this review is to investigate the key contributors to disease burden in patients with AAV and to describe strategies for improving health-related quality of life.

## Graphical abstract

**Figure:**
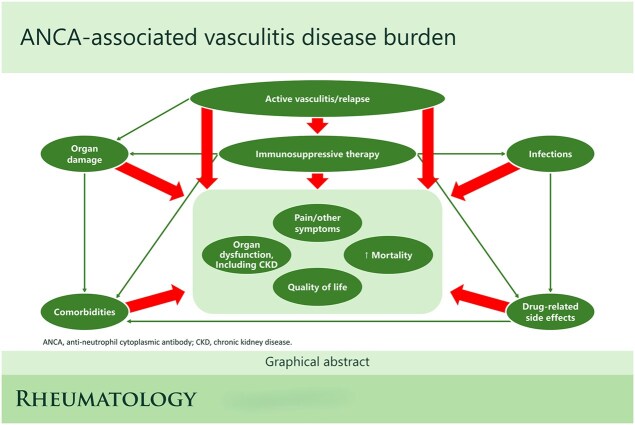

Rheumatology key messages

- AAV disease burden includes organ impairment, inflammation-related comorbidities, treatment-related adverse effects, relapse, and reduced survival.
- Rituximab reduces the risk of AAV relapse, organ damage and comorbidities in AAV patients.
- Reducing glucocorticoid exposure improves quality of life and reduces treatment-related adverse effects.

## Introduction

Anti-neutrophil cytoplasmic antibody (ANCA)-associated vasculitis (AAV) is a group of autoimmune vascular disorders which includes granulomatosis with polyangiitis (GPA), microscopic polyangiitis (MPA), and eosinophilic granulomatosis with polyangiitis (EGPA) [1, 2]. Early AAV is characterized by the signs and symptoms of chronic inflammatory diseases, such as fatigue, weight loss, myalgia, and polyarthralgia. As the disease progresses, patients are at risk of developing vasculitis-related organ impairment and comorbidities associated with chronic inflammation, including diabetes mellitus, hypertension, and cardiovascular diseases (CVD). The aims of treatment are to reduce the risk of organ damage by rapidly controlling active disease and preventing AAV relapse [3, 4]. This is achieved using immunosuppression-based therapies [typically rituximab (RTX) or cyclophosphamide (CYC) alongside glucocorticoids (GC)] and avacopan [3–5]. Although effective, immunosuppression is associated with a high risk of adverse events (AE) (particularly infections) and drug-related organ damage/comorbidities. The overall consequences of AAV and AAV treatments include limited survival, organ impairment, chronic pain and multiple other symptoms, many of which contribute to a reduced health-related quality of life (HRQoL). The aim of this review is to investigate the key factors contributing to disease burden in patients with GPA/MPA and to describe strategies for improving HRQoL.

## AAV disease burden

### Mortality

Prior to the advent of immunosuppression-based induction therapy in the late 1950s, the average time from diagnosis to death in patients with GPA was ∼5 months, with some patients dying in as little as 4 weeks [6]. At this time, most patients (∼75%) died within 4 years, and the primary causes of death were kidney failure (57%), respiratory failure (24%), and cardiac failure (21%), with ∼5% of patients dying from infections [6]. The introduction of immunosuppressive induction therapy has considerably improved survival rates, with data from seven European Vasculitis Study Group (EUVAS) clinical trials reporting an estimated median survival of 17.8 years (95% CI 15.7–20.0 years) among 848 patients with GPA/MPA followed for a median of 8 years [interquartile range (IQR) 2.9–13.6 years] [7]. However, patients continue to experience limited survival compared with the general population, with excess cumulative mortality ranging from ∼14% at 1 year to 20% at 10 years and 29% at 15 years [7]. Advanced age, male gender, a low estimated glomerular filtration rate (eGFR) and platelet counts <250 × 10^9^/l were strong baseline predictors of death among patients receiving immunosuppression-based therapy, and the main causes of death were treatment-related infections (26% of deaths over a median 8 years), CVD (14%), and malignancies (13%) [7]. This suggests a need for newer therapies which effectively control active disease while reducing the risk of these endpoints.

### AAV relapse

AAV relapse (defined as a recurrence of active AAV after a period of remission [3]) is a major contributor to disease burden, affecting ∼30–50% of patients over 5 years [8]. Relapse rates vary across clinical trials depending on patient characteristics and treatment strategies, with the lowest rates (∼10–15% over 2–3 years) reported in patients receiving RTX-based maintenance therapies [3, 9].

Risk factors for AAV relapse include a GPA diagnosis, PR3-ANCA positivity, lower levels of serum creatinine (SCr), more aggressive disease, and baseline complications related to the ENT [8, 9]. Risk is also increased in patients following RTX withdrawal [10], and in those with lower levels of CYC exposure [10], a history of AAV relapse [9, 11], ANCA positivity at the end of induction therapy [9, 11], and (although not confirmed for patients receiving RTX-based treatment in MAINRITSAN [8] and RITAZAREM [9] clinical trials) an increase in ANCA levels during maintenance therapy [9, 11]. This suggests that patients with risk factors for AAV relapse might benefit from longer, more intensive RTX-based maintenance therapy than those without.

### Infections

Infections are the main causes of death among patients with AAV, both during the early phase of therapy and after long-term follow-up [7]. Pooled data from seven EUVAS trials (*N* = 848) found that infections accounted for 46.2% of deaths in patients with new-onset AAV during the first year of follow-up and remained high (19.0%) after >5 years [7]. Consistent with these data, a cohort study in 549 patients with incident AAV reported that, compared with healthy age- and sex-matched controls, AAV patients experienced a 77-fold higher risk of developing severe infections during the first 30 days of treatment (i.e. during periods of aggressive immunosuppressive therapy) [odds ratio (OR) 77.02 (95% CI 11.67–508.6)], decreasing to a 3.8-fold increased risk over a period of 16 years [12].

*Post hoc* analysis of RAVE trial data (*N* = 197) found that the most common types of severe infection in patients receiving RTX- or CYC-based induction therapy for GPA/MPA were respiratory tract infections (RTIs) (68.2% of infections), followed by urinary tract infections (UTIs) and gastrointestinal (GI) infections (4.5% each) [13]. The risk of severe RTIs was considerably higher among patients treated with CYC (81.8%) *vs.* RTX (54.5%), whereas the risk of severe UTIs and severe GI infections was higher among patients receiving RTX (9.1% each) *vs.* CYC (0% each).

**Figure 1.**
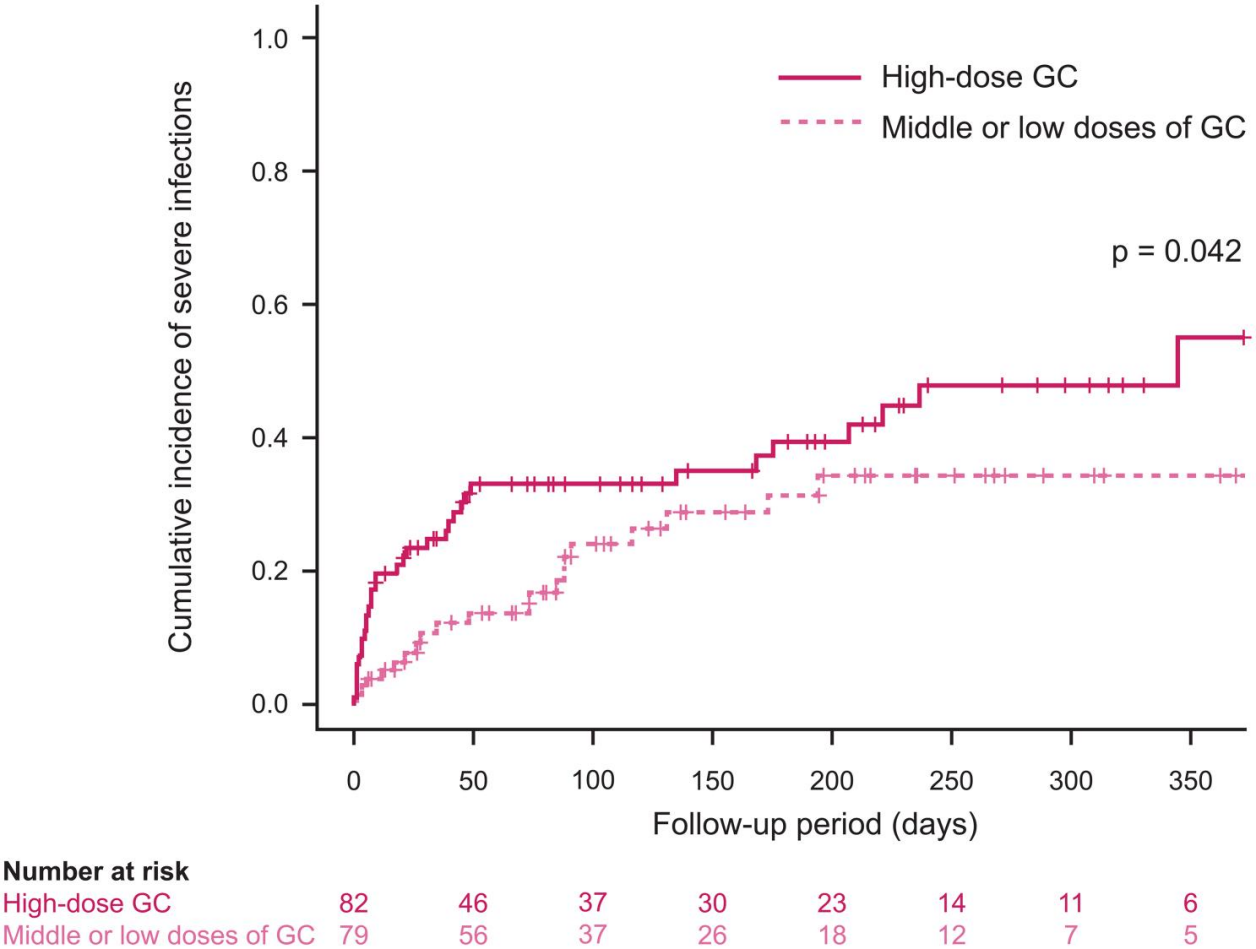
Correlation between GC dose and cumulative incidence of severe infection. Cumulative incidences of severe infections were significantly higher in patients receiving initial high-dose GC, considering death without severe infections as a competing risk (*P* = 0.042). GC: glucocorticoid. Figure reproduced from Waki D et al. Initial high-dose corticosteroids and renal impairment are risk factors for early severe infections in elderly patients with Anti-neutrophil cytoplasmic autoantibody-associated vasculitis: a retrospective observational study. Medicine (Baltimore) 2020;99:e19173, with kind permission from Wolters Kluwer Health, Inc.

Risk factors for severe infections include low levels of CD19+ B cells (especially among patients treated with RTX), high levels of serum immunoglobulin M, older age, and kidney involvement at baseline [13, 14], with some studies [14] (but not others [13]) suggesting a correlation between infection risk and baseline disease activity, assessed using the BVAS [13]. In addition, an observational study in 167 elderly patients (≥65 years) with AAV found that the cumulative incidence of severe infections was significantly higher in patients receiving high-dose GC (prednisolone ≥0.8 mg/kg/day) *vs.* middle- or low-dose GC (Fig. 1) [15]. Patients with early severe infections exhibited a high mortality rate within 6 months of treatment, suggesting a possible relationship between infection-related deaths and early GC exposure. Consistent with these results, a study in 114 patients with new-onset severe AAV (defined as SCr >500 μmol/l or dialysis dependency) reported that the addition of intravenous methylprednisolone to induction therapy was associated with a higher risk of infection during the first 3 months of treatment [hazard ratio (HR) 2.7 (95% CI 1.4–5.3); *P* = 0.004] but had little or no impact on survival, kidney recovery or relapse rates [16]. Two recent clinical trials have demonstrated that faster GC taper had similar efficacy compared with the previous standard, but was associated with a significantly reduced risk of infection [17, 18]. This suggests it might be possible to reduce the risk of infections (at least in some patients) by reducing early GC exposure.

In addition to reducing GC exposure, the risk of infections can be reduced using trimethoprim-sulfamethoxazole and (in patients with repeated infections and hypogammaglobulinemia) intravenous immunoglobulin G replacement therapy (IVIG). The effects of trimethoprim-sulfamethoxazole in patients with AAV were demonstrated by a *post hoc* analysis of data from the RAVE trial (*N* = 197), in which low-dose trimethoprim-sulfamethoxazole significantly reduced infections including but not limited to pneumocystis pneumonia by up to 80% when administered alongside induction therapy [13]. The effects of IVIG on infection risk were demonstrated by a retrospective cohort study in 142 patients with hypogammaglobulinemia (IgG <7 g/dl) following RTX treatment, 101 of which (71%) had AAV [19]. In this study, the risk of hypogammaglobulinemia increased in patients receiving RTX for >2 years and continued to increase with longer durations of RTX treatment. Risk factors for hypogammaglobulinemia (immunoglobulin G < 5 g/l) and/or IVIG use at 60 months included prior CYC exposure [OR 3.60 (95% CI 1.03–12.53)], GC use at 12 months [OR 7.48 (95% CI 1.28–43.55)], low nadir levels of IgG [OR 0.68 (95% CI 0.51–0.90)], and female sex [OR 8.57 (95% CI 2.07–35.43)] [19]. However, the severity of hypogammaglobulinema did not directly correlate with the risk of infection [19]. Consequently, IVIG is only recommended for the treatment of hypogammaglobulinemia in patients with repeated infections.

### Organ damage

Combined data from six EUVAS trials following 302 patients with newly diagnosed AAV for a mean 7.3 years found that the most frequent consequences of vasculitis damage in people with GPA and MPA were impaired kidney function (eGFR <50 ml/min/1.73 m^2^), hypertension, and proteinuria followed by nasal blocking/crusting, hearing loss, peripheral neuropathy, osteoporosis, end-stage kidney disease (ESKD), impaired pulmonary function and malignancy [20]. Low eGFR (affecting 60% of MPA patients; 31.7% of GPA patients), proteinuria (48.1% MPA; 34.1% GPA) and ESKD (20% MPA; 9% GPA) were significantly more common in patients with MPA *vs* GPA, whereas nasal blocking/crusting (4.4% MPA; 44.3% GPA) and hearing loss (4.4% MPA; 32.3% GPA) were more common in patients with GPA. In this study that had been conducted in the pre-rituximab era, 47.8% of patients were receiving GCs at their last study visit, the mean duration of GC treatment was 40.4 ± 16.7 months, and almost 30% of patients received GCs for 60 months. This suggests that the rates of vasculitis damage in this study were, in part, related to the intense use of GCs.

The impact of vasculitis damage on HRQoL in patients with AAV is particularly high in patients with ESKD, many of whom require regular dialysis. In the EUVAS trials, 175 of the 848 patients with GPA/MPA (21%) developed ESKD and required kidney replacement therapy after a median follow-up of 7.96 years (IQR 2.95–13.64), of which 140 (80%) received dialysis and 35 (20%) received a kidney transplant [21]. The chances of regaining kidney function are considerably greater during the early stages of kidney disease. Consequently, patients with kidney impairment should be identified early. A multivariate Cox regression model found that risk factors associated with ESKD include advanced age (>65 years) [HR 1.68 (95% CI 1.18–2.41); *P* = 0.005], lower baseline eGFR [HR 0.97 (95% CI 0.93–0.99); *P* < 0.001] and lower baseline levels of hemoglobin [HR 0.88 (95% CI 0.78–0.98); *P* = 0.02]. This suggests a need to closely monitor patients with these risk factors for early changes in kidney function.

### Comorbidities

A population-based study of patients with AAV diagnosed between 1998 and 2010 found that, compared with the general population (*n* = 744), people with AAV (*n* = 186) had a higher prevalence of diabetes mellitus [rate ratio (RR) 2.0 (95% CI 1.3–2.9); *P* = 0.003], hypertension [RR 1.4 (95% CI 1.1–1.8); *P* = 0.02], ischemic heart disease [RR 1.5 (95% CI 1.0–2.3); *P* = 0.07] and myocardial infarction [RR 2.0 (95% CI 1.0–3.6); *P* = 0.06], but significantly lower rates of dyslipoproteinemias [RR 0.6 (95% CI 0.3–1.1); *P* = 0.04] and little or no difference in cerebrovascular accident [RR 1.1 (95% CI 0.6–2.0); *P* = 0.7] [22]. In addition, AAV was associated with a significantly increased prevalence of venous thrombosis [RR 4.0 (95% CI 1.9–8.3); *P* = 0.003), osteoporosis [RR 4.6 (95% CI 3.0–7.0); *P* < 0.001], and thyroid diseases [RR 2.1 (95% CI 1.3–3.3); *P* = 0.009].

### Other GC-related adverse events

**Figure 2.**
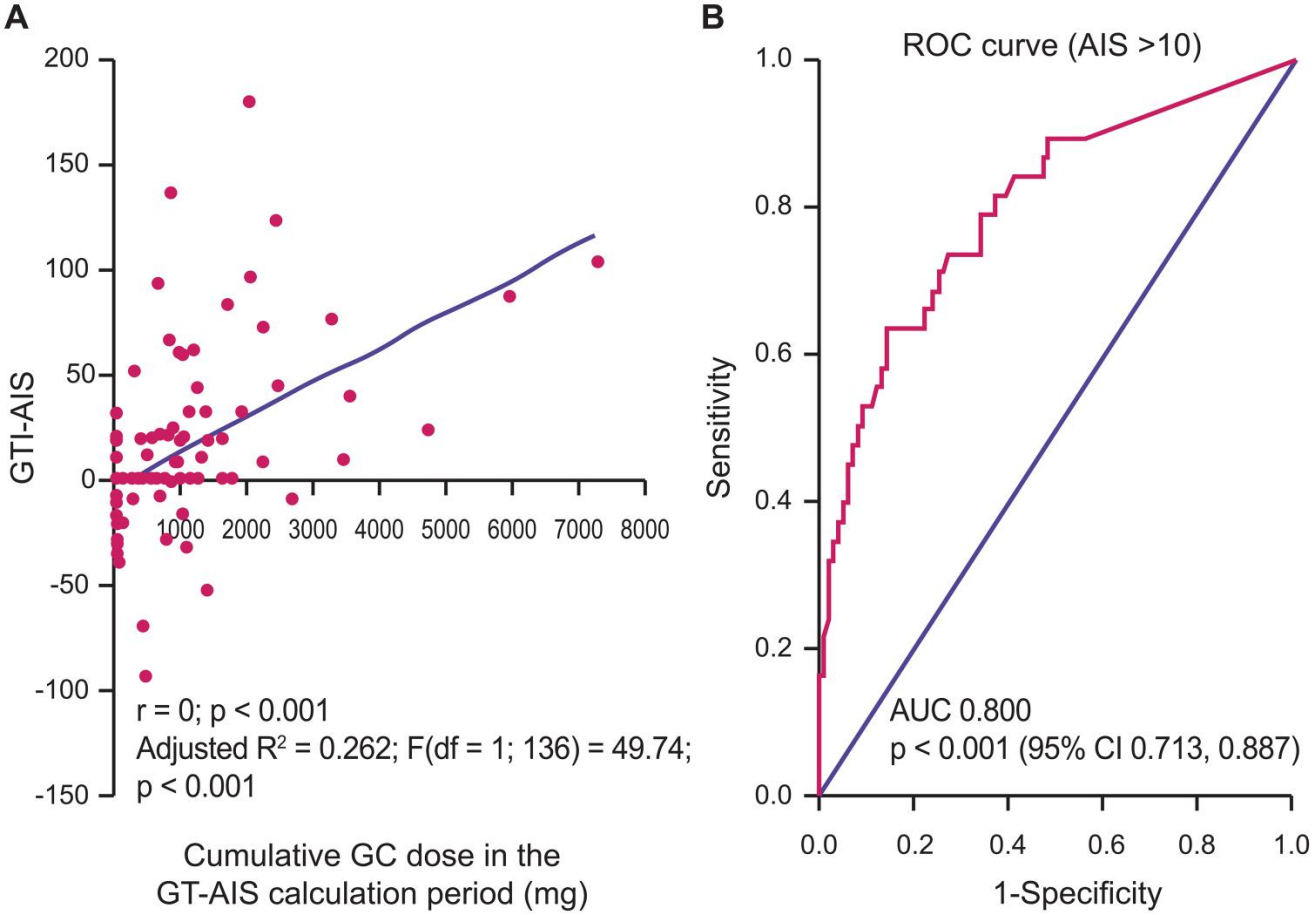
Correlation between cumulative GC dose and GTI-AIS (**A**) and the likelihood of a clinically meaningful change in GTI-AIS (**B**). (**A**) Scatter plot showing the relationship between cumulative GC dose in mg and changes in GTI-AIS from baseline to 6 months. (**B**) ROC curve to determine the cumulative GC threshold dose between t1 and t2 beyond which GTI-AIS is >10. AIS: Aggregate Improvement Score; AUC: area under the curve; GC: glucocorticoids; GTI: Glucocorticoid Toxicity Index; ROC: receiver operating characteristics. Figures reproduced from Scherbacher PJ et al. Prospective study of complications and sequelae of glucocorticoid therapy in ANCA-associated vasculitis. RMD Open 2024;10:e003956, with permission from BMJ Publishing Group; permission conveyed through Copyright Clearance Center, Inc.

In addition to the risk of infection discussed above, drug-related AEs can have a major impact on overall disease burden. A prospective study of 138 patients with AAV [mean total cumulative GC dose 9014 mg (IQR 4974.8–17,389.3); median disease duration 57 months (IQR 25.8–103.5)] found a significant correlation between GC exposure and Glucocorticoid Toxicity Index-Aggregate Improvement Score (GTI-AIS) (Fig. 2A), with patients receiving cumulative GC doses >935 mg having an 80% likelihood of a clinically meaningful change in GTI-AIS (Fig. 2B) [23]. The most frequent GC-related AEs varied depending on whether the patient received short-term, high-dose GCs for active disease or long-term, lower-dose GCs as part of maintenance therapy. Overall, 37.9% of patients with active disease developed diabetes or glucose intolerance, 31.0% reported sleep disturbance, and 20–30% had weight gain, hypertension and/or infections, while patients in remission most frequently experienced skin atrophy (38.5%).

## Health-related quality of life

**Figure 3.**
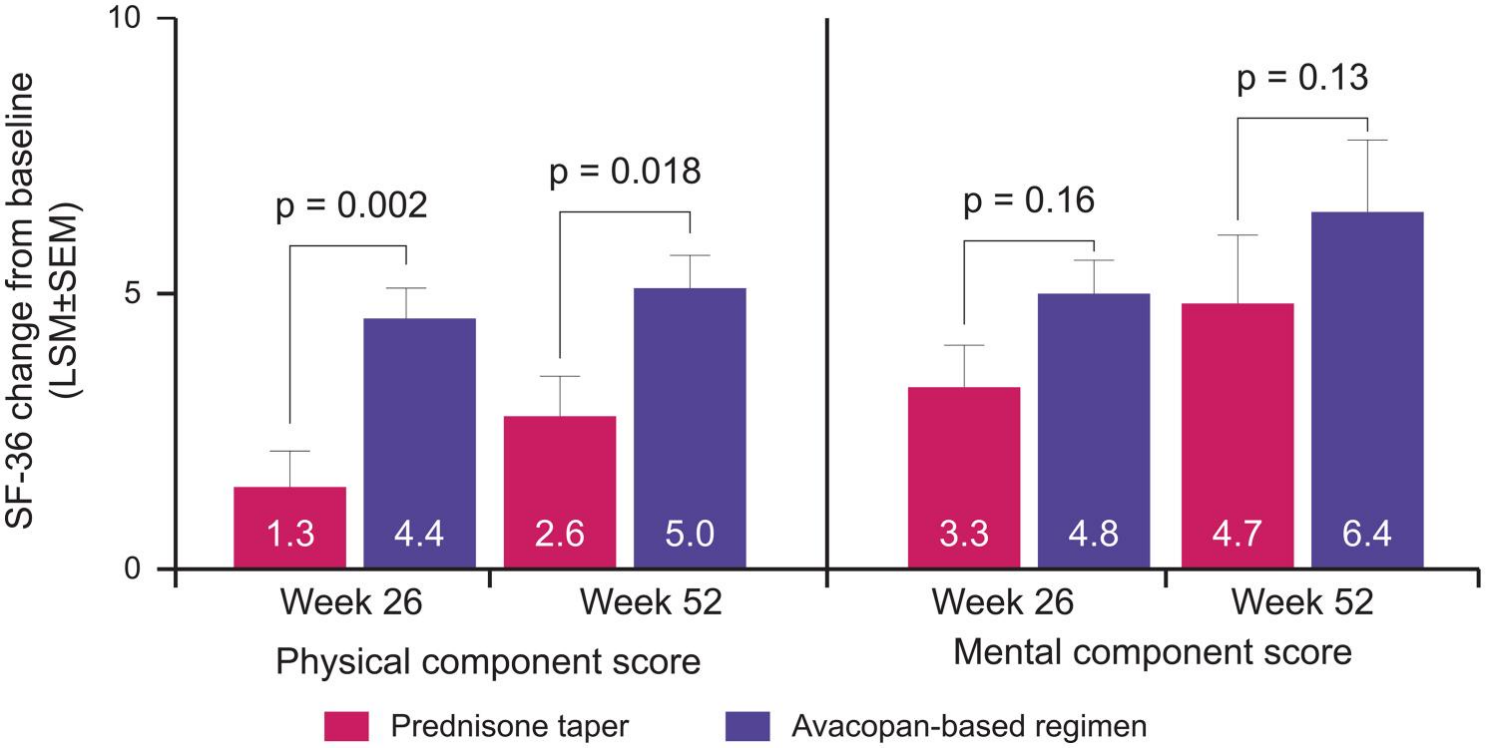
Change from baseline in SF-36 physical and mental component summary scores at weeks 26 and 52 among ADVOCATE trial patients receiving avacopan *vs*. prednisone taper. Patient numbers at weeks 26 and 52 were 147 and 144, respectively, in the prednisone taper group. Patient numbers at weeks 26 and 52 in the avacopan group were 153 and 147, respectively (physical component summary score), and 154 and 148, respectively (mental component summary score) [24]. GC: glucocorticoid; LSM: least squares mean; SF-36: 36-Item Short Form Health Survey version 2

HRQoL is often impaired in patients with AAV, with many patients (especially those receiving long-term, high-dose GC) reporting depression, anxiety, employment difficulties, fatigue and pain [24]. *Post hoc* analysis of patient-reported outcomes (PRO) data from the phase 3 ADVOCATE trial (*N* = 331) demonstrated HRQoL improvements from baseline to weeks 26 and 52 in patients receiving standard therapy (RTX or CYC + GC) for GPA or MPA [24] (Fig. 3). Improvements in physical component score and mental component score were greater at both timepoints in patients achieving early GC reductions through the adjunctive use of avacopan, with improvements in mental health largely being driven by improvements in emotional and vitality domains [24]. This highlights the benefits of early avacopan use enabling rapid GC tapering in patients with AAV.

Changes in HRQoL during the ADVOCATE trial were assessed using the 36-item Short Form Health Survey [SF-36] version 2, the EuroQoL 5-dimension 5-level (EQ-5D-5L) questionnaire, and the EQ-5D health utility measure [24]. Although useful, none of these tools was specifically designed for use in patients with AAV. To further investigate the effects of AAV on HRQoL, a new tool (AAV-PRO) was developed that captures PROs specific to AAV, including organ-specific symptoms, systemic-symptoms, treatment-related AEs, social and emotional impact, concerns about the future and physical function [25]. A monocentric, prospective longitudinal study in 156 patients with AAV identified a clear correlation between levels of pain, depression, functional limitations and patient perceptions of disease activity, with only a weak correlation between AAV-PRO scores and clinician-based measures, including BVAS and Vascular Damage Index. This supports the inclusion of AAV-PRO as a validated outcome measure in clinical trials and as a communication tool in clinical practice.

## Summary and conclusions

AAV outcomes have considerably improved since the introduction of RTX-based treatment strategies and reduced-dose GC regimens [17, 26, 27]. However, effective treatment is limited by a number of challenges, including limited survival compared with the general population [7], high rates of infection-related deaths irrespective of the duration of follow-up [7], high rates of relapse affecting between 30% and 50% of patients over 5 years [8], the risk of treatment-related (in particular GC-related) acute and chronic comorbidities [23], and the high risk of organ damage, including ESKD [21]. Each of these challenges has an impact on patient function and HRQoL [24, 25]. This suggests a need for more effective and well tolerated therapies that enable GC dose reductions in patients with new-onset and relapsing AAV.

## Data Availability

No new data were generated or analysed in support of this article.

## References

[1] Kronbichler A , Lee KH, Denicolò S et al Immunopathogenesis of ANCA-associated vasculitis. Int J Mol Sci 2020;21:7319. 33023023 10.3390/ijms21197319 PMC7584042

[2] Almaani S , Fussner LA, Brodsky S, Meara AS, Jayne D. ANCA-associated vasculitis: an update. J Clin Med 2021;10:1446. 33916214 10.3390/jcm10071446 PMC8037363

[3] Hellmich B , Sanchez-Alamo B, Schirmer JH et al EULAR recommendations for the management of ANCA-associated vasculitis: 2022 update. Ann Rheum Dis 2024;83:30–47. 36927642 10.1136/ard-2022-223764

[4] Kidney Disease: Improving Global Outcomes AVWG. KDIGO 2024 Clinical Practice Guideline for the Management of Antineutrophil Cytoplasmic Antibody (ANCA)-associated vasculitis. Kidney Int 2024;105:S71–116. 38388102 10.1016/j.kint.2023.10.008

[5] Hellmich B. Mapping a path forward: addressing disease burden, pathways and solutions in ANCA-associated vasculitis. Rheumatology (Oxford). 2025;keaf517. 10.1093/rheumatology/keaf517 PMC12783590 41051263

[6] Walton EW. Giant-cell granuloma of the respiratory tract (Wegener’s granulomatosis). Br Med J 1958;2:265–70. 13560836 10.1136/bmj.2.5091.265 PMC2026251

[7] Sánchez Álamo B , Moi L, Bajema I et al; EUVAS. Long-term outcomes and prognostic factors for survival of patients with ANCA-associated vasculitis. Nephrol Dial Transplant 2023;38:1655–65. 36617233 10.1093/ndt/gfac320

[8] King C , Druce KL, Nightingale P et al Predicting relapse in anti-neutrophil cytoplasmic antibody-associated vasculitis: a systematic review and meta-analysis. Rheumatol Adv Pract 2021;5:rkab018. 34476335 10.1093/rap/rkab018 PMC8407598

[9] Salama AD. Relapse in anti-neutrophil cytoplasm antibody (ANCA)-associated vasculitis. Kidney Int Rep 2020;5:7–12. 31922056 10.1016/j.ekir.2019.10.005 PMC6943777

[10] King C , Harper L. Avoidance of harm from treatment for ANCA-associated vasculitis. Curr Treatm Opt Rheumatol 2017;3:230–43. 29201630 10.1007/s40674-017-0082-y PMC5694500

[11] Al-Soudi A , Vegting Y, Klarenbeek PL, Hilhorst ML. Do relapses follow ANCA rises? A systematic review and meta-analysis on the value of serial ANCA level evaluation. Front Med (Lausanne) 2022;9:844112. 35860735 10.3389/fmed.2022.844112 PMC9289208

[12] Zhao K , Dehghan N, Xie H, Esdaile J, Aviña-Zubieta JA. Increased risk of severe infection in patients with antineutrophil cytoplasmic antibody-associated vasculitides: a population-based trend analysis. Ann Rheum Dis 2024;83:182–3. 10.1007/s10067-026-08096-w 41949711

[13] Odler B , Riedl R, Gauckler P et al; RAVE-ITN Research Group. Risk factors for serious infections in ANCA-associated vasculitis. Ann Rheum Dis 2023;82:681–7. 36702528 10.1136/ard-2022-223401 PMC10176387

[14] Rathmann J , Jayne D, Segelmark M, Jönsson G, Mohammad AJ. Incidence and predictors of severe infections in ANCA-associated vasculitis: a population-based cohort study. Rheumatology (Oxford) 2021;60:2745–54. 33253372 10.1093/rheumatology/keaa699

[15] Waki D , Nishimura K, Tokumasu H et al Initial high-dose corticosteroids and renal impairment are risk factors for early severe infections in elderly patients with antineutrophil cytoplasmic autoantibody-associated vasculitis: a retrospective observational study. Medicine (Baltimore) 2020;99:e19173. 32080098 10.1097/MD.0000000000019173 PMC7034627

[16] Chanouzas D , McGregor JAG, Nightingale P et al Intravenous pulse methylprednisolone for induction of remission in severe ANCA associated vasculitis: a multi-center retrospective cohort study. BMC Nephrol 2019;20:58. 30777023 10.1186/s12882-019-1226-0 PMC6378728

[17] Walsh M , Merkel PA, Peh C-A et al; PEXIVAS Investigators. Plasma exchange and glucocorticoids in severe ANCA-associated vasculitis. N Engl J Med 2020;382:622–31. 32053298 10.1056/NEJMoa1803537 PMC7325726

[18] Furuta S , Nakagomi D, Kobayashi Y et al; LoVAS Collaborators. Effect of reduced-dose vs high-dose glucocorticoids added to rituximab on remission induction in ANCA-associated vasculitis: a randomized clinical trial. JAMA 2021;325:2178–87. 34061144 10.1001/jama.2021.6615 PMC8170547

[19] Tieu J , Smith RM, Gopaluni S et al Rituximab associated hypogammaglobulinemia in autoimmune disease. Front Immunol 2021;12:671503. 34054846 10.3389/fimmu.2021.671503 PMC8149951

[20] Robson J , Doll H, Suppiah R et al Damage in the anca-associated vasculitides: long-term data from the European vasculitis study group (EUVAS) therapeutic trials. Ann Rheum Dis 2015;74:177–84. 24243925 10.1136/annrheumdis-2013-203927

[21] Sachez-Alamo B , Moi L, Bajema I et al Long-term outcome of kidney function in patients with ANCA-associated vasculitis. Nephrol Dial Transplant 2024;39:1483–93. 38268409 10.1093/ndt/gfae018 PMC11361807

[22] Englund M , Merkel PA, Tomasson G, Segelmark M, Mohammad AJ. Comorbidities in patients with antineutrophil cytoplasmic antibody-associated vasculitis versus the general population. J Rheumatol 2016;43:1553–8. 27252425 10.3899/jrheum.151151

[23] Scherbacher PJ , Hellmich B, Feng YS, Loffler C. Prospective study of complications and sequelae of glucocorticoid therapy in ANCA-associated vasculitis. RMD Open 2024;10:e003956. 38428978 10.1136/rmdopen-2023-003956 PMC10910690

[24] Strand V , Jayne DRW, Horomanski A et al; ADVOCATE Study Group. The impact of treatment with avacopan on health-related quality of life in antineutrophil cytoplasmic antibody-associated vasculitis: a *Post hoc* analysis of data from the ADVOCATE trial. Lancet Rheumatol 2023;5:e451–60. 38251577 10.1016/S2665-9913(23)00092-9

[25] Maunz A , Jacoby J, Henes J et al Association of the AAV-PRO questionnaire with established outcome measures in AAV. Rheumatology (Oxford) 2024;63:174–80. 37129542 10.1093/rheumatology/kead199

[26] Treppo E , Binutti M, Agarinis R, De Vita S, Quartuccio L. Rituximab induction and maintenance in ANCA-associated vasculitis: state of the art and future perspectives. J Clin Med 2021;10:3773. 34501224 10.3390/jcm10173773 PMC8432126

[27] Miloslavsky EM , Specks U, Merkel PA et al; Rituximab in ANCA-Associated Vasculitis-Immune Tolerance Network Research Group. Rituximab for the treatment of relapses in antineutrophil cytoplasmic antibody-associated vasculitis. Arthritis Rheumatol (Hoboken, NJ) 2014;66:3151–9. 10.1002/art.38788 PMC4229846 25047592

